# Impacts of different methods of conception on the perinatal outcome of intrahepatic cholestasis of pregnancy in twin pregnancies

**DOI:** 10.1038/s41598-018-22387-6

**Published:** 2018-03-05

**Authors:** Chun Feng, Wen-Juan Li, Rong-Huan He, Xi-Wen Sun, Guirong Wang, Li-Quan Wang

**Affiliations:** 1 The Second Affiliated Hospital, School of Medicine, Zhejiang University, Hangzhou, Zhejiang, 310009 China; 2 The Women’s Hospital, School of Medicine, Zhejiang University, Hangzhou, Zhejiang, 310006 China; 3 Department of Surgery, SUNY Upstate Medical University, Syracuse, New York, 13210 USA

## Abstract

Twin pregnancies have a higher prevalence of intrahepatic cholestasis of pregnancy (ICP) than single pregnancies. It is unknown whether *in vitro* fertilization-embryo transfer (IVF-ET) influences the fetal outcomes in twin pregnancies complicated by ICP. This study aimed to explore the impact of IVF-ET on the perinatal outcomes of ICP in twin pregnancy. Clinical data from 142 twin pregnant women complicated with ICP were retrospectively analyzed, including 51 patients who conceived through IVF-ET (IVF group) and 91 patients with spontaneous conception (SC group). Several biochemical indicators and perinatal outcomes were analyzed. Compared to the SC group, the IVF group had a higher incidence of early-onset ICP (*P* = 0.015) and more frequent clinical symptoms (*P* = 0.020), including skin pruritus, skin scratch, and jaundice. Furthermore, the IVF group had higher rates of neonatal asphyxia (IVF vs. SC, 9.80% vs. 1.10%, *P* = 0.023) and premature delivery (IVF vs. SC, 96.08% vs. 83.52%, *P* = 0.027) compared to the SC group. The IVF-conceived twin pregnancy group had a higher risk of early-onset ICP and suffered from clinical symptoms and poor perinatal outcomes.

## Introduction

Intrahepatic cholestasis of pregnancy (ICP) is a reversible pregnancy-associated liver disease that is characterized by elevated serum bile acids and an increased risk of deleterious consequences for the fetus, especially in the late second or third trimester^1–3^. Clinical symptoms triggered by ICP can influence the quality of life of pregnant women, whereas the maternal outcome is usually benign. A higher frequency of fetal complications, such as preterm labor, intrauterine fetal distress, and sudden intrauterine fetal demise, is associated with ICP^3–6^. Given the multiple factors involved in the pathogenesis of ICP, detailed mechanisms are not fully understood. A genetic susceptibility to the cholestatic effect of reproductive hormones and their metabolites may be involved due to an impaired bile secretory function in susceptible women^7^. For example, mutations in ABCB11 caused enhanced susceptibility to ICP^8,9^ and the lack of ABCB4 gene product resulted in defective functioning of the phospholipid export pump and impaired biliary secretion of phosphatidylcholine^10^. Variants or mutations of the genes controlling hepatobiliary transport or related nuclear regulators may play a role in the occurrence and/or severity of ICP^11^. Placental transfer of bile acids may be impaired in ICP patients, and there is a reversed transplacental gradient of bile acids^12^. In addition, unidentified environmental factors such as observed geographical and seasonal variations of ICP may also contribute to the etiology^11^. For more than 30 years, it has been widely accepted that ICP is related to a genetically predisposed abnormal reaction of the maternal liver to estrogen^13^. Recent studies have indicated that estradiol (E_2_) might induce ICP by repressing the expression of the bile salt export pump (BSEP)^14^ or by up-regulating the PXR signaling pathway^15^.

Our previous study found that the serum E_2_ levels of women who conceived through *in vitro* fertilization-embryo transfer (IVF-ET) at 4 and 8 weeks of gestation were significantly higher than those of women who conceived naturally^16^. Further studies found that E_2_ and progesterone levels were elevated in the umbilical blood of IVF-conceived children^17^, suggesting that the high estrogen environment persists throughout the entire gestational period. In view of the influence of estrogen on ICP, the incidence of ICP was found to be elevated in IVF-conceived pregnancies compared to SC pregnancies^18,19^.

A previous study from Chile found that twin pregnancies had a higher prevalence of ICP (20.9%) than single pregnancies (4.7%)^20^. Furthermore, increased incidences of meconium staining of amniotic fluid, LBW, and preterm birth have been observed in twin pregnancies with ICP, as well as stillbirth earlier in pregnancy in twins versus singletons^21^. Since assisted reproductive technique (ART) treatment increases the risk of adverse maternal complications of ICP^18,19^, we asked the following questions: Does IVF-ET influence the incidence of ICP in twin pregnancies? How is the fetal outcome in twin pregnancies complicated by ICP? To address these questions, this study analyzed and compared the clinical features and fetal outcomes in twin pregnancies with ICP between IVF and SC pregnancies. Moreover, early-onset ICP was limited to ICP with an onset time of <28 gestational weeks. Adverse fetal outcomes affected 35.1% women with early-onset and 21.3% with late onset and included LBW, fetal morbidity and mortality, especially in the early-onset group^22^. Early-onset ICP has a severe consequence on the prognosis of offspring; therefore, we used it as a key parameter in this study.

## Materials and Methods

**Figure 1:**
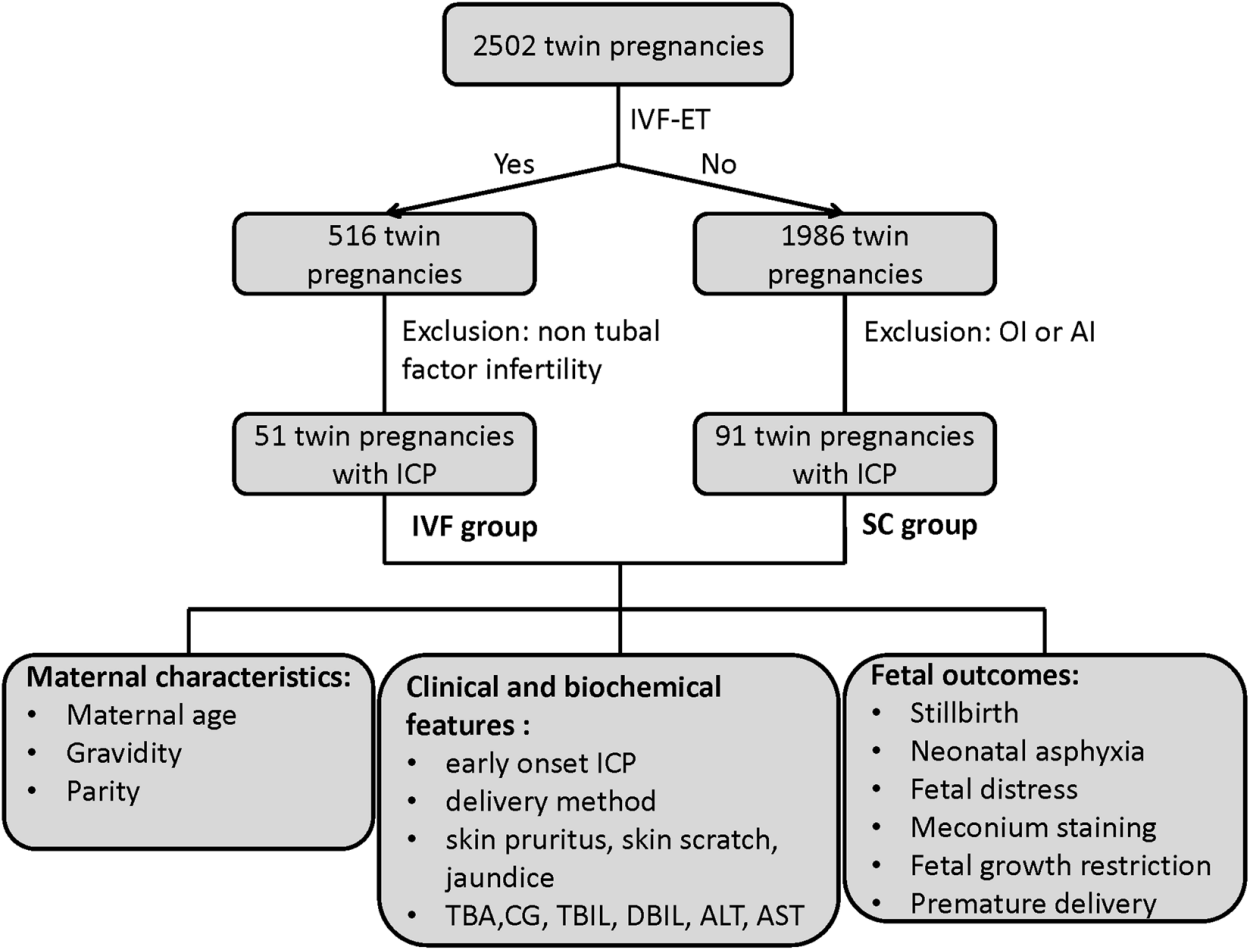
Flow chart of the present study. A total of 2502 twin pregnancies were reviewed, including 516 conceived through IVF-ET and 1986 conceived spontaneously (SC). Ultimately, 51 patients conceived through IVF-ET (IVF group) and 91 patients conceived spontaneously (SC group) suffered from intrahepatic cholestasis of pregnancy (ICP).

This study was a retrospective analysis of data from 142 twin pregnant women complicated with ICP. All of the patients were delivered in the Women’s Hospital School of Medicine Zhejiang University, Hangzhou, Zhejiang Province, China. As shown in Fig. 1, a total of 2502 twin pregnancies were delivered from January 2003 to February 2012, including 516 conceived through IVF and 1986 conceived naturally. To avoid the interference of immune factors, sexual hormones and other underlying diseases from infertile couples, we included IVF-pregnant women with tubal factor infertility exclusively to minimize uncontrollable bias, and pregnancies conceived through ovulation induction (OI) or artificial insemination (AI) were excluded from the spontaneously conceived cases. The exclusion criteria were as follows: (1) infertility factors except tubal factor infertility; (2) pregnancies after OI or AI; (3) any of the following causes of liver dysfunction: viral or autoimmune hepatitis, acute fatty liver of pregnancy, primary biliary cirrhosis, preeclampsia, hemolysis, elevated liver enzymes and low platelets (HELLP) syndrome, and other hepatic imaging abnormalities. Finally, 142 twin pregnant women complicated with ICP were included: 51 patients who conceived through IVF-ET due to tubal factor infertility (IVF group) and 91 patients with spontaneous conception (SC group).

During pregnancy, a routine prenatal examination was performed every four weeks before 28 gestational weeks and every two weeks after 28 gestational weeks. The chorionicity was determined by an ultrasound performed between 7 and 14 gestational weeks. Serum bile acids (SBA) were detected whenever the patients had pruritus, and all other patients had routine SBA tests for the first time at 24 gestational weeks. The criteria for diagnosis of ICP were elevated levels of maternal SBA (total bile acid level ≥10 μmol/L) with or without pruritus without itching skin disease and quick disappearance after delivery, according to the guidelines formulated by the obstetric group of the Chinese Medical Association’s Obstetrics and Gynecology branch.

SBA was detected by enzymatic cycling assay on Abbott ARCHITECT i2000 with Total Bile Acid Diagnostic Reagent (AusBio Laboratories Co., Ltd.). The imprecision was ≤15%, and the measuring range was 5 μmol/L-180 μmol/L. Serum cholyglycine (CG) concentration was examined using chemiluminescent immunoassay (CLIA) on Maglumi 2000 Chemiluminescence Immunoassay System with CG *in vitro* diagnostic kits. The imprecision was ≤ 10%, and the measuring range was 2.5 μg/dL – 4000 μg/dL.

When patients were diagnosed with ICP, all eligible subjects received drug therapy of ursodeoxycholic acid (UDCA) and regular prenatal examinations until delivery. The clinical features of pregnant women, including skin pruritus, skin scratch, jaundice, and other biochemical indicators, such as total bile acid, alanine aminotransferase (ALT), and aspartate aminotransferase (AST), were recorded at the time of diagnosis (before treatment). Furthermore, fetal outcomes, including fetal distress (Apgar score of less than 7 at 5 minutes), neonatal asphyxia (Apgar score ≤7 at 5 minutes, fetal blood gas of pH < 7.2 and acid-base <−14 to −16 mmol/L, with no other factors leading to a low Apgar score), stillbirth, premature delivery, meconium staining, birth weight, and birth weight centile (the national birth weight centiles by gestational age for Chinese twins as reference^23^) were recorded and studied. Any ICP cases diagnosed before the third trimester (<28 weeks) were considered early-onset. Severe ICP was diagnosed with the following criteria: ① SBA ≥ 40 μmol/L; ② severe pruritus; ③ complicated with multiple pregnancy, pregnancy-induced hypertension syndrome, recurrent ICP, or previous perinatal infant death due to ICP; ④ early-onset ICP^24^. The criteria for mild ICP included the following: ① SBA ≥10 μmol/L and <40 μmol/L and ② no manifestation other than pruritus^24^. So all the patients in our study suffered severe ICP. Pregnancy was terminated during 38–39 gestational weeks for mild ICP and during 34–37 gestational weeks for severe ICP. Delivery was induced when fetal distress was indicated or treatment received no response. Generally, cesarean section was performed for a twin pregnancy with ICP. This study was approved by the Ethical Committee of the Women’s Hospital, School of Medicine, Zhejiang University, and all methods were performed in accordance with the relevant guidelines and regulations. Since the design was a retrospective study, informed consent was not obtained. As data were anonymised, the risk of identification was minimal.

Statistical analysis was performed using SPSS version 19.0 for windows (IBM, Amonk, NY, USA). Quantitative data were expressed as the mean ± SD and analyzed using a two-tailed t-test. The numerical data were compared using a chi-square test or Fisher’s exact test. Adjusted odds ratios (OR) with 95% confidence intervals (CI) were calculated to assess the risks of early-onset ICP, neonatal asphyxia, and premature delivery. The ORs were adjusted for maternal age, parity (1, ≥2), and chorionicity using binary logistic regression. The levels of serum bile acids were divided as 10–20, 20–30, 30–40, ≥40 µM, and the CG concentrations as <1000, 1000–2000, 2000–3000, ≥3000 µg/dL. Then the neonatal complications were compared among different groups with binary logistic regression. Statistical significance was accepted when P value <0.05.

## Results

The incidence of ICP was higher in twin pregnancies conceived through IVF than in those conceived naturally (9.88% vs. 4.58%, *P* < 0.0001). A total of 142 twin pregnant women with ICP who met the criteria described in the materials and methods section were included in the present study. As shown in Table 1, maternal age and the incidence of dichorionic twins were higher and parity was lower in the IVF group than in the SC group (*P* < 0.05). There were no differences between the two groups in terms of gravidity or delivery method (*P* > 0.05). Of the 142 patients, 2 from the IVF group and 15 from the SC group had previously delivered, but none of them had a history of ICP. Both IVF and SC patients were classified as having mild ICP when diagnosed.

**Table 1:** Maternal Characteristics of IVF and spontaneous conception (SC) groups.

Items		IVF group (N = 51)	SC group (N = 91)	P value
Maternal age		30.37 ± 2.65	29.17 ± 3.73	0.028*
Gravidity	1	26 (50.98%)	52 (57.14%)	0.479
	≥2	25 (49.02%)	39 (42.86%)	
Parity	1	49 (98.08%)	76 (83.52%)	0.027*
	≥2	2 (3.92%)	15 (16.48%)	
Chorionicity	monochorionic	3 (5.88%)	22 (24.18%)	0.006*
	dichorionic	48 (94.12%)	69 (75.82%)	
Early-onset ICP	<28 w	12 (23.53%)	9 (9.89%)	0.015*
	≥28 w	39 (76.47%)	82 (90.11%)	
Delivery method	CS	51 (100%)	88 (96.70%)	0.553
	VD	0 (0%)	3 (3.30%)	

The data were presented as mean ± SD. *P *<* 0.05.

IVF, *in vitro* fertilization; CS: cesarean section; VD, Vaginal delivery.

A higher incidence of ICP occurred before 28 weeks of pregnancy in the IVF group than in the SC group (23.53% vs. 9.89%, *P* = 0.015). Considering the differences in maternal age, parity and chorionicity between the two groups, ORs adjusted for these three factors were calculated. The most associated factor of early-onset ICP was found to be IVF, with an adjusted OR of 4.98 (95% CI: 1.53–16.28, *P* = 0.008). Additionally, ICP-related clinical symptoms, including skin pruritus, skin scratch, and jaundice, were more common in the IVF group than in the SC group (*P* = 0.020). However, there were no significant differences between the two groups in ICP and/or liver function-related biochemical factors when diagnosed (Table 2).

**Table 2:** Biochemical features in twin pregnant women when diagnosed.

Items	IVF group (N = 51)	SC group (N = 91)	P value
TBA (μmol/l)	20.51 ± 18.68	20.03 ± 19.88	0.890
CG (μg/dl)	2296.96 ± 1845.44	2072.34 ± 2651.40	0.596
TBIL (μmol/l)	10.51 ± 7.48	10.65 ± 5.66	0.905
DBIL (μmol/l)	3.94 ± 5.75	4.07 ± 4.20	0.884
ALT (U/L)	54.55 ± 80.43	60.19 ± 92.90	0.719
AST (U/L)	51.49 ± 73.51	54.78 ± 70.20	0.796

The data were presented as mean ± SD. TBA, total biliary acid; CG, cholyglycine; TBIL, total bilirubin; DBIL, direct bilirubin; ALT, alanine aminotransferase; AST aspartate aminotransferase.

The patients had not yet received the treatment of ursodeoxycholic acid (UDCA) when these parameters were detected.

We further analyzed fetal outcomes in the two groups. The results showed that the IVF group had higher incidences of neonatal asphyxia (IVF vs. SC, 9.80% vs. 1.10%, *P* = 0.023) and premature delivery (IVF vs. SC, 96.08% vs. 83.52%, *P* = 0.027) than the SC group (Table 3). Twin pregnancies conceived through IVF were more commonly associated with neonatal asphyxia, with an OR of 45.84 (95% CI: 2.41–873.69, *P* = 0.011) adjusted for premature delivery, chorionicity, and early-onset ICP. The risk of premature delivery was increased with IVF, with an OR of 5.07 (95% CI: 1.07–23.93, *P* = 0.041) adjusted for chorionicity and early-onset ICP. Although the incidence of fetal distress showed an elevated tendency in the IVF group, no significant differences were observed in stillbirth, meconium staining, or cesarean section between the two groups (Table 3). The birth weight in the IVF group was significantly lower than that in the SC group (IVF vs. SC, 2167.92 ± 375.03 g vs. 2269.51 ± 391.95 g, *P* = 0.033). However, the incidences of birth weight <10^th^ Centile and cases >90^th^ Centile were similar between the two groups.

**Table 3:** Fetal outcomes in twin pregnant women.

Items	IVF group (N = 51)	SC group (N = 91)	P value	Adjusted P value
Stillbirth	1 (1.96%)	0 (0%)	0.359	—
Neonatal asphyxia	5 (9.80%)	1 (1.10%)	0.023^*^	0.011^*^
Fetal distress	7 (13.72%)	6 (6.59%)	0.224	—
Meconium staining	6 (11.76%)	6 (6.59%)	0.350	—
<10^th^ Centile	8 (7.92%)	15 (8.24%)	1.000	—
>90^th^ Centile	5 (4.95%)	7 (3.85%)	0.760	—
Premature delivery	49 (96.08%)	76 (83.52%)	0.027^*^	0.041^*^

^*^P < 0.05.

**Figure 2:**
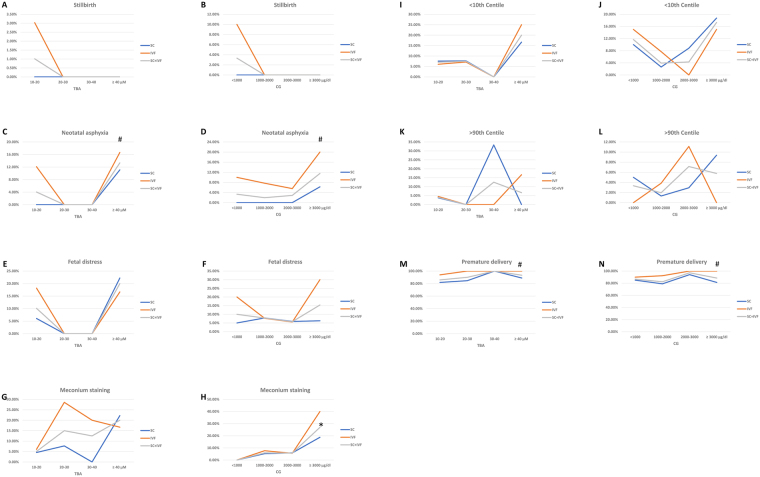
Correlation of bile acids levels and neonatal complications. (**A,C,E,G,I,K** and **M**). The correlation between TBA and stillbirth, neonatal asphyxia, fetal distress, meconium staining, birth weight <10^th^ centile, birth weight >90^th^ centile, and premature delivery. (**B,D,F,H,J,L** and **N**). The correlation between CG and stillbirth, neonatal asphyxia, fetal distress, meconium staining, birth weight <10^th^ centile, birth weight >90^th^ centile, and premature delivery. SC: spontaneous conception. IVF: *in vitro* fertilization and ET. TBA: total bile acids. CG: cholyglycine. *P < 0.05 correlated with TBA or CG. ^#^P < 0.05 compared between SC and IVF.

The relationship between ranges of serum bile acids levels and the incidence of neonatal complications was analyzed, as shown in Fig. 2. Although there was an increased trend of incidences of neonatal complications while TBA or CG increased, however, no statistical significance was achieved of the correlation between neonatal complications and TBA levels (*P* > 0.05). The incidence of meconium staining was correlated to CG concentration with OR = 3.012 (95% CI: 1.446–6.278) (*P* = 0.003), while all the other complications were not correlated to CG concentration (*P* > 0.05).

## Discussion

The safety aspects of ART for mother and fetus have been a concern since the introduction of the technology more than 30 years ago, and much of the risk is due to the increase of multiple gestations, which have increased risks for prematurity, perinatal mortality and morbidity^25^. The present study has addressed the impact of IVF on the perinatal outcome of twin pregnancy with ICP.

Evidence of the effect of method of conception on pregnancy outcome in twin pregnancies is controversial. A comparison between 104 ART twin and 193 non-ART pregnancies showed that, in the ART group, the incidences of pregnancy-induced hypertension, uterine bleeding, premature contractions, intrauterine growth retardation, fetal death, discordance, and cesarean section were significantly higher, birth weight of both twins was lower, more were admitted to the neonatal intensive care unit (NICU), and more second twin neonates died^26^. A series of studies in later indicated similar risks^27–32^. But some studies showed different observations. Perinatal outcomes did not differ between pregnancies conceived through assisted reproductive technology and conceived naturally^33^. No increased risks of preterm birth or NICU admission were found in IVF/ICSI conceived twins, although IVF/ICSI conceived twins were more likely to be delivered by CS and have RDS and neonatal hypoglycaemia^34^. IVF/ICSI conception was not correlated with birth weight, Apgar score, pregnancy-related disorders^35^. Considering none of these studies were about the twin pregnancies with ICP, we focused on the perinatal outcome of twin pregnancies complicated with ICP in this study and compared our findings with those of previously published work about twin pregnancies.

In the present study, a tendency of early-onset ICP was found in the IVF group compared to the SC group. A majority of ICP patients are diagnosed in the late second or third trimester of pregnancy, but a few cases have been diagnosed as early as 8 weeks^36^. Compared with late-onset ICP (onset time ≥28 gestational weeks), early-onset ICP presented worse clinical manifestations (higher incidence of premature delivery, meconium staining, fetal distress, newborn asphyxia, developing into severe ICP, and cesarean section)^37^ and higher TBA, TBIL^37^, aminotransferase activity, and bilirubin concentrations^38,39^. In twin pregnancy, clinical manifestations might be more serious^40^. Given the poor prognosis of early-onset ICP, the increased risk of early-onset ICP in IVF-conceived twin pregnancy could lead to serious outcomes. Therefore, it is important to explore the underlying mechanisms.

A systematic review found that the risk of admission to a neonatal intensive care unit increased with relative risk of 1.05 (95% CI 1.01 to 1.09)^41^. In the present study IVF group had higher rates of neonatal asphyxia (IVF vs. SC, 9.80% vs. 1.10%), indicating that in twins complicated with ICP the risk of neonatal asphyxia was higher, and this might lead to an increased risk of admission to NICU.

Previously systematic review demonstrated that preterm twins differed widely in frequency of premature delivery (IVF vs. SC, 18.8–60.0% vs. 20.0–52.4%), and the relative risk of preterm birth was 1.07 (95% CI 1.02 to 1.13) in IVF group^41^. In our study the incidence of premature delivery increased in IVF group compared to SC group (IVF vs. SC, 96.08% vs. 83.52%). We found that the risk of premature delivery was higher in twins complicated with ICP, but the tendency was coherent. That means pregnancies conceived with IVF tended to be premature delivery, no matter with or without ICP.

As mentioned above, ovarian hormones and genetic factors are important in the pathogenesis of ICP. In animal studies, estrogens have been shown to reduce bile acid uptake by hepatocytes and cause cholestasis^42^. Progesterone administration in the third trimester of pregnancy might induce the development of ICP, and the metabolites of progesterone may be higher in patients with ICP than in controls^43,44^. During IVF-ET, controlled ovarian hyperstimulation (COH) situates the gamete/embryo in a supra-physiological ovarian hormone environment^17,45^, which may increase the morbidity of ICP and result in an increased risk of early-onset ICP in IVF-conceived pregnancies.

Of the biochemical markers for ICP, bile acid is the most suitable for the diagnosis and prediction of fetal risk using the cholic acid level. The early onset of raised serum bile acid was an independent predictor of preterm delivery^2,46^. In the present study, although the IVF group had more cases of early-onset ICP than the SC group, no differences were observed in TBA or serum CG levels between the IVF and SC groups. With ICP, the onset of raised AST and ALT may occur either before or after elevated serum bile acids^47,48^, and the risk factors differ between early-onset (serum bile acid and ALP) and late-onset (ALP and gamma-glutamyl transpeptidase) ICP^22^.

In this study, different fetal outcomes of twin pregnancies between IVF and SC groups were found, demonstrating that the IVF group had higher rates of neonatal asphyxia and premature delivery than the SC group. We found that even if adjusted for early-onset ICP and chorionicity, IVF increased the risk of premature delivery with an OR of 5.07. The following factors may increase the incidence of premature delivery: (1) patients conceived through IVF tend to be more anxious and are more fragile facing the risk of babies; (2) ICP occurs earlier in IVF patients, which increases the mental burden of both patients and doctors. Therefore, when we treat twin pregnancies complicated with ICP after IVF, we should be calm and avoid increasing the incidence of indicated preterm birth.

More premature deliveries may partly explain the higher incidence of neonatal asphyxia in the IVF group. However, IVF increased the risk of asphyxia after the adjustment for premature delivery, indicating that additional factors were involved. The cause of poorer outcomes in IVF seems to be multifactorial. Altered endocrine profiles changed by controlled ovarian hyperstimulation (COH) in IVF at an early stage might influence implantation and placental development. The subfertility itself, including parental characteristics such as endocrine disorders, abnormal immune system function, and older age, may interact with other factors^49^.

We found that the incidence of meconium staining was correlated to CG concentration. Similar results were observed in a recent published work, in which the risk of meconium staining was significantly higher when ICP was diagnosed or delivered at earlier gestational ages, or when TBA concentrations were higher at diagnosis^50^.

In the present study only TBA was examined, so endogenous major bile acids and UDCA could not be discriminated. It was reported that treatment of ICP with UDCA could decrease more than half of the endogenous serum bile acid level, reduce pruritus, and bring down ALT and bilirubin, but did not benefit for neonatal complications, such as preterm delivery, neonatal asphyxia, and meconium staining^51–53^. The duration of UDCA treatment was correlated positively with neonatal asphyxia in ICP, which might be related with the longer exposure to high bile acids level^54^. It is necessary to address this aspect in the future study, through using mass spectrometry. That will allow us examine serum concentrations of endogenous bile acids change during UDCA treatment and gain information about the relationship of these parameters of the mother and the neonatal outcome.

In summary, the present study indicated that IVF-conceived twin pregnancies have an increased risk of developing early-onset ICP, and suffer from poor fetal outcomes, especially premature delivery and neonatal asphyxia, more frequently than spontaneously conceived twin pregnancies. Hence, early and accurate diagnosis, appropriate medical intervention, and closer monitoring are mandatory for improving fetal prognosis, especially in IVF-conceived twin pregnancies. Meanwhile, the determination of pregnancy termination should be done with care in pregnancies conceived through IVF to control the incidence of indicated preterm birth.
